# Isolated Hepatic Splenosis: First Reported Case

**DOI:** 10.1155/1998/72067

**Published:** 1998-08

**Authors:** Michael D'Angelica, Yuman Fong, Leslie H. Blumgart

**Affiliations:** 1 Hepatobiliary Service Department of Surgery Memorial Sloan-Kettering Cancer Center USA; 2 Department of Surgery Memorial Sloan-Kettering Cancer Center York Ave. New York NY 1275 USA

## Abstract

Splenosis is the autotransplantation of splenic tissue, most commonly seen after traumatic splenic rupture and splenectomy. Post-traumatic splenosis is often considered a rare entity, but is probably underreported because of its asymptomatic nature. We describe the first reported case of splenosis presenting as a liver mass, indistinguishable from a liver tumor by standard preoperative evaluation. The pathophysiology, evaluation and management of splenosis is discussed as well as the decision to resect a benign appearing liver mass.


*HPB Surgery*, 1998, Vol. 11, pp. 39–42
Reprints available directly from the publisher
Photocopying permitted by license only

© 1998 OPA (Overseas Publishers Association) N.V.
Published by license under
the Harwood Academic Publishers imprint,
part of The Gordon and Breach Publishing Group.
Printed in India.

### *Case Report*

### Isolated Hepatic Splenosis: First Reported Case

MICHAEL D'ANGELICA, YUMAN FONG and LESLIE H. BLUMGART*

*Hepatobiliary Service, Department of Surgery, Memorial Sloan-Kettering Cancer Center*

*(Received 10 June 1997)*

**Splenosis is the autotransplantation of splenic tissue, most commonly seen after traumatic splenic rupture and splenectomy. Post-traumatic splenosis is often considered a rare entity, but is probably underreported because of its asymptomatic nature. We describe the first reported case of splenosis presenting as a liver mass, indistinguishable from a liver tumor by standard preoperative evaluation. The pathophysiology, evaluation and management of splenosis is discussed as well as the decision to resect a benign appearing liver mass.**

*Keywords:* Splenosis, liver mass, liver resection

### INTRODUCTION

Splenosis, the autotransplantation of splenic tissue, is a well documented phenomena after splenic rupture and splenectomy. Nodules are characteristically multiple, diffusely spread throughout the peritoneal cavity and appear as small reddish blue nodules. Post traumatic splenosis is generally considered an uncommon event, but is probably under reported because of its asymptomatic nature. Splenic implants have been shown, both experimentally and clinically, to be able to implant anywhere they are spread at the time of trauma [1]. We present the first reported case of splenosis presenting as an isolated liver mass, indistinguishable from a liver tumor by standard preoperative evaluation. The clinical presentation of this entity will be reviewed and the importance of a high index of suspicion for this diagnosis in patients with a history of splenic trauma emphasized.

### CASE REPORT

The patient is a 38 year old female with a history of alcohol use and intermittent oral contraceptive use who was found to have abnormal liver enzymes on a screening test performed by her primary care physician. She had no history of weight loss, abdominal pain or jaundice. Her past medical history was significant for a distal pancreatectomy and splenectomy after a motor vehicle accident 20 years prior to admission. She

*Corresponding author. Present address: Department of Surgery, Memorial Sloan-Kettering Cancer Center, 1275 York Ave., New York, NY.

denied any history of smoking and reported drinking six cans of beer a day. There was no significant family history of malignancy.

Physical examination revealed a well developed, healthy appearing middle aged female with normal vital signs. Sclera were anicteric. Abdominal exam revealed a long left paramedian scar, a transverse left upper quadrant scar and a right lower quadrant scar with no palpable masses or hepatosplenomegaly. There was no abdominal tenderness or evidence of ascites. The remainder of the exam was within normal limits.

Serum chemistries were significant for glucose of 147 mg/dl (65–115), alkaline phosphatase of 231 U/L (25–140), AST of 288 U/L (0–40), ALT of 161 U/L (0–45), total bilirubin of 1.4 mg/dl (0.1–1.2), and a GGTP of 2490 U/L (0–85). Serologies for Hepatitis A, B and C were negative.

An abdominal ultrasound was performed that revealed diffusely increased echogenicity in the liver consistent with hepatocellular disease, and a hypoechoic lesion felt to be in the left liver measuring 3.6 × 3.9 cm. Absence of the spleen was confirmed. A CT scan of the abdomen was then obtained (Fig. 1) and showed hepatomegaly with diffusely decreased density throughout the liver consistent with fatty infiltration. A rounded, well circumscribed high density mass was seen in the anterior aspect of the liver straddling the medial and lateral segments of the left liver, measuring 3.2×3.6 cm. No central scar in the mass was appreciated. The etiology of the mass was felt to be focal nodular hyperplasia or an adenoma.

The patient desired surgical removal of this mass because of associated bleeding risks and was referred to us. She was taken to the operating room where extensive adhesions from her previous trauma were noted. There were also dense adhesions between the liver in the area of the tumor and the undersurface of the diaphragm. The tumor was found to be approximately 2.5 cm in diameter, located within the umbilical fissure and to be approximately 2.5 cm in diameter, located within the umbilical fissure and occupying part of segments 3 and 4. The rest of the abdominal exploration was unremarkable. The tumor was subsequently enucleated and the operative diagnosis by gross inspection (Fig. 2) was an adenoma. The patients post operative recovery was uneventful. Histologic examina-

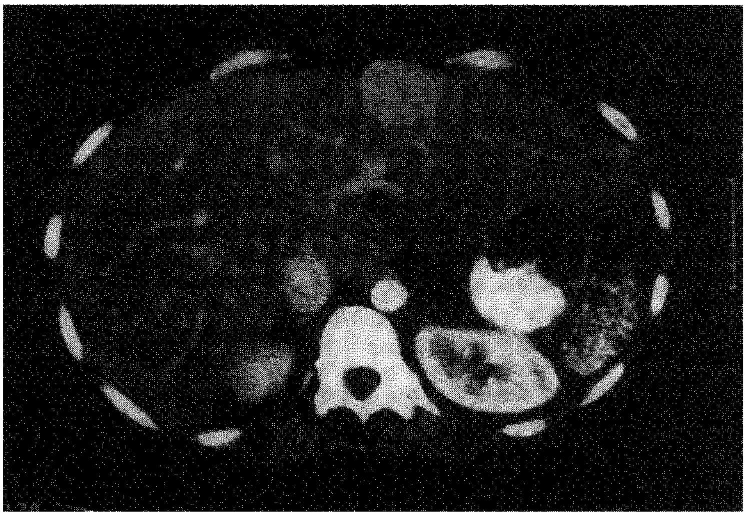

FIGURE 1 Abdominal computed tomography scan revealing a 3.2×3.6 cm high density mass in the anterior aspect of the left lobe of the liver.

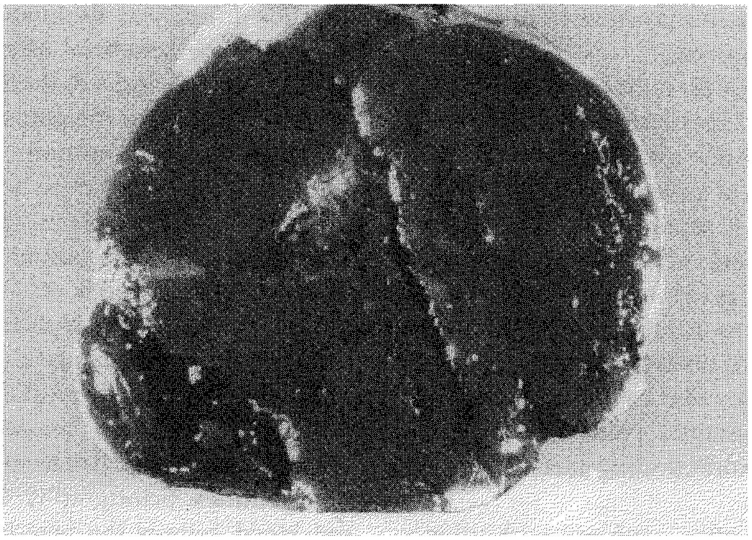

FIGURE 2 Gross pathological specimen indistinguishable from a hepatic adenoma.

tion of the mass revealed splenic tissue attached to the liver surface with unremarkable liver and splenic parenchyma.

### DISCUSSION

Splenosis is caused by the implantation of splenic fragments onto exposed vascularized surfaces at the time of splenic trauma. The blood supply to these splenic implants is derived from small penetrating vessels arising from donor surfaces. The implants are usually small and multiple with as many as hundreds throughout the peritoneal cavity. The fragments can implant anywhere to which they have access, but are most commonly found on the serosal surfaces of the small intestine, greater omentum, mesentery, undersurface of the diaphragm and in the pelvis [1]. There have been reports of thoracic splenosis following diaphragmatic rupture and subcutaneous splenosis following gunshot wounds through the spleen [1,2]. Splenic implants mimicking renal masses [3] and intestinal masses [1] have also been reported. There have been two previous reports of splenic implants on the surface of the liver, however they were incidental findings at autopsy [4]. Presumably, for a splenic nodule to implant in the liver, there must be a simultaneous rupture of the liver capsule at the time of trauma. This case represents the first report of an antemortem diagnosis of hepatic splenosis and emphasizes the need to consider this diagnosis in a patient with a history of splenic trauma.

We report a patient who presented with abnormal liver enzymes and a hepatic mass that was clinically consistent with a adenoma or focal nodular hyperplasia. Operatively, the mass was felt to be an adenoma. The patient also had a significant histroy of alcohol use and this may have been the cause of her laboratory abnormalities, Since hepatic adenomas have a significant risk of hemorrhage and are potentially premalignant, a resection was performed. This case emphasizes the need to consider splenosis in the differential diagnosis of an unexplained hepatic mass in patients who have a history of splenic trauma. Although, this is the first report of a splenic nodule presenting as an isolated hepatic mass, if splenosis was considered preoperatively, the patient may have been spared a laparotomy and resection.

Splenosis was once thought to be uncommon, but the incidence of splenosis following trauma or surgery is probably underreported because the large majority of cases are asymptomatic. Recently it has been reported in 26 to 67% of patients with traumatic rupture of the spleen [3]. The most common presentation of splenosis is an incidental finding at laparotomy or laparoscopy for unrelated disease. Rarely, splenosis will become symptomatic as a cause of bowel obstruction or abdominal pain, but there are usually associated confounding factors in these cases [1].

The entities most often confused with splenosis at laparotomy are metastatic cancer, endometriosis, hemangiomas and accessory spleens. Differentiating these diagnoses can be difficult. It is important to understand the difference between splenosis and accessory spleens. In contrast to splenosis, accessory spleens are congenital, usually limited in number (rarely more than six), are typically located along the splenopancreatic ligament, are supplied by a branch of the splenic artery and have a true hilus [4]. Although, the splenic implant in this case was singular, it lacked a hilus, followed splenic trauma and was anatomically inconsistent with an accessory spleen. We are therefore convinced that this is a case of splenosis.

The immunologic value of the spleen is well documented and it is known that susceptibility to overwhelming infection is much higher in the asplenic host. Interestingly, it has been shown that splenic nodules left behind after splenectomy retain splenic filtering and immunologic function [5]. Given its innocuous nature, splenosis may be beneficial after traumatic splenic splenic rupture. Indeed, there is no indication to remove splenosis if the patient is asymptomatic.

Because most remnant splenic tissue is asymptomatic and may be beneficial to the host, preoperative diagnosis is desirable. However, splenosis is rarely encountered by the physician and not often thought of. The most important principle is to have a high level of suspicion in patients with unexplained masses and a history of splenic trauma. Typical imaging modalities used for abdominal masses scuh as CT or ultrasound will not differentiate splenosis from other entities. Scintigraphy with heat damaged red blood cells is the best test to obtain if the diagnosis is considered [2]. Indeed if an unexplained mass is proven to be splenic tissue and the patient lacks any symptoms from it, splenosis should not be resected. This report widens the spectrum of presentation for splenosis and adds the liver as a potential site of its presentation.

### *References*

[1] Fleming, C. R., Dickson, E. R. and Harrison, E. G. (1976). Splenosis Autotransplantation of Splenic Tissue. *Am. J. Med.*, **61**, 414–419.

[2] Hibbeln, J. F., Wilbur, A. C., Schreiner, V. C. and Trepashko, D. W. (1995). Subcutaneous Splenosis: *Clin. Nucl. Med.*, **20**, 591–593.

[3] Bock, D. B., King, B. F., Hezmall, H. P. and Oesterling, J. E. (1991). Splenosis Presenting as a Left Renal Mass Indistinguishable from Renal Cell Carcinoma. *J. Urol*, **146**, 152–154.

[4] Cohen, E. A. (1954). Splenosis. *Arch. Surg.*, **69**, 777–784.

[5] Hathaway, J. M., Harley, R. A., Self, S., Schiffman, G. and Virella, G. (1995). Immunologic Function in Post-traumatic Splenosis. *Clin. Immunol. Immunopathol.*, **74**, 143–150.

